# External validation, update and development of prediction models for pre-eclampsia using an Individual Participant Data (IPD) meta-analysis: the International Prediction of Pregnancy Complication Network (IPPIC pre-eclampsia) protocol

**DOI:** 10.1186/s41512-017-0016-z

**Published:** 2017-10-03

**Authors:** John Allotey, Kym I. E. Snell, Claire Chan, Richard Hooper, Julie Dodds, Ewelina Rogozinska, Khalid S. Khan, Lucilla Poston, Louise Kenny, Jenny Myers, Basky Thilaganathan, Lucy Chappell, Ben W. Mol, Peter Von Dadelszen, Asif Ahmed, Marcus Green, Liona Poon, Asma Khalil, Karel G. M. Moons, Richard D. Riley, Shakila Thangaratinam, for the IPPIC Collaborative Network

**Affiliations:** 1 Women’s Health Research Unit, Barts and the London School of Medicine and Dentistry, Queen Mary University of London, London, UK; 2 Pragmatic Clinical Trials Unit, Barts and the London School of Medicine and Dentistry, Queen Mary University of London, London, UK; 3 Multidisciplinary Evidence Synthesis Hub (MESH), Queen Mary University of London, London, UK; 4 Research Institute for Primary Care and Health Sciences, Keele University, Keele, UK; 5 Division of Women’s Health, Women’s Health Academic Centre, King’s College London, London, UK; 6 Irish Centre for Fetal and Neonatal Translational Research [INFANT], University College Cork, Cork, Ireland; 7 Maternal and Fetal Heath Research Centre, Manchester Academic Health Science Centre, University of Manchester, Central Manchester NHS Trust, Manchester, UK; 8 Fetal Medicine Unit, St George’s University Hospitals NHS Foundation Trust and Molecular and Clinical Sciences Research Institute, St George’s University of London, London, UK; 9 The Robinson Research Institute, School of Paediatrics and Reproductive Health, University of Adelaide, Adelaide, Australia; 10 Institute of Cardiovascular and Cell Sciences, St George’s, University of London, London, UK; 11 Aston Medical School, Aston University, Birmingham, UK; 12 Action on Pre-eclampsia (APEC) Charity, Worcestershire, UK; 13 Harris Birthright Research Centre for Fetal Medicine, King’s College Hospital, London, UK; 14 Department of Obstetrics and Gynaecology, The Chinese University of Hong Kong, Hong Kong, Hong Kong; 15 Julius Centre for Health Sciences and Primary Care, University Medical Centre Utrecht, Utrecht, the Netherlands

**Keywords:** Pre-eclampsia, Prognosis, Prediction model, Maternal, Fetal, IPD, Individual participant data

## Abstract

**Background:**

Pre-eclampsia, a condition with raised blood pressure and proteinuria is associated with an increased risk of maternal and offspring mortality and morbidity. Early identification of mothers at risk is needed to target management.

**Methods/design:**

We aim to systematically review the existing literature to identify prediction models for pre-eclampsia. We have established the International Prediction of Pregnancy Complication Network (IPPIC), made up of 72 researchers from 21 countries who have carried out relevant primary studies or have access to existing registry databases, and collectively possess data from more than two million patients. We will use the individual participant data (IPD) from these studies to externally validate these existing prediction models and summarise model performance across studies using random-effects meta-analysis for any, late (after 34 weeks) and early (before 34 weeks) onset pre-eclampsia. If none of the models perform well, we will recalibrate (update), or develop and validate new prediction models using the IPD. We will assess the differential accuracy of the models in various settings and subgroups according to the risk status. We will also validate or develop prediction models based on clinical characteristics only; clinical and biochemical markers; clinical and ultrasound parameters; and clinical, biochemical and ultrasound tests.

**Discussion:**

Numerous systematic reviews with aggregate data meta-analysis have evaluated various risk factors separately or in combination for predicting pre-eclampsia, but these are affected by many limitations. Our large-scale collaborative IPD approach encourages consensus towards well developed, and validated prognostic models, rather than a number of competing non-validated ones. The large sample size from our IPD will also allow development and validation of multivariable prediction model for the relatively rare outcome of early onset pre-eclampsia.

**Trial registration:**

The project was registered on Prospero on the 27 November 2015 with ID: CRD42015029349.

## Background

Pre-eclampsia, a condition with raised blood pressure and proteinuria in pregnancy, remains a leading cause of maternal deaths worldwide [1], and is one of the commonest causes of maternal admission to intensive care in high-income countries [2]. It is associated with increased perinatal mortality and foetal growth restriction, and contributes to 10% of stillbirths and 15% of preterm births [3, 4]. When pre-eclampsia occurs before 34 weeks’ gestation, known as early onset disease, it considerably increases the risk of maternal complications, with a 20-fold higher maternal mortality than late onset disease [5–7].

Quantifying a woman’s risk of developing pre-eclampsia during the course of the pregnancy is important to guide clinical decisions and monitoring strategies. Pregnant women at high risk of pre-eclampsia require close monitoring, and should be started on prophylactic aspirin to reduce adverse outcomes [8]. Early commencement of this has the potential for maximum benefit [8]. Currently, clinical assessment for risk of pre-eclampsia is mainly based on clinical history [9]; however, such risk-based predictions have shown limited accuracy. In recent years, there has been intensive interest in developing prediction models that incorporate additional tests for biochemical and ultrasound markers to improve predictive performance [10, 11]. Early onset disease, occurring before 34 weeks’ gestation, is more severe, and is considered to have a different pathophysiology than the late onset disease. It is unlikely that a single model will accurately predict both early and late onset disease [12].

Clinical applicability of the tests and models predicting pre-eclampsia based on the findings of aggregate meta-analyses is limited. This is due to the observed heterogeneity in populations, in the combinations of predictors and in outcome definitions (e.g. most published models focussed on any pre-eclampsia rather than the much more clinically severe early onset pre-eclampsia); and by the lack of robust methods for aggregating data of published models. Furthermore, prior to the use of prediction models in clinical practice, there is a need to successfully validate the model in multiple datasets external to the model development data. This often takes many years to accomplish in a primary study.

Individual Participant Data (IPD) meta-analysis can overcome many of the above limitations by accessing the raw data of the individual participants. A large scale IPD meta-analysis will enable us to predefine the desired clinically relevant endpoints (e.g. timing of pre-eclampsia onset). It will allow us to standardise the definitions of predictors and outcomes, take into account the performance of many candidate prognostic variables, directly handle missing data on both predictors and outcomes, account for heterogeneity in baseline risks, and most importantly, develop, validate and tailor the use of the most accurate prediction models to the appropriate population [13].

## Methods/design

The IPPIC project will be undertaken using existing recommendations on prognostic research model development and validation [14–16], and by adhering to recent reporting guidelines for prediction models and IPD meta-analysis [17, 18]. The project is registered on the International prospective register of systematic reviews (Prospero) with registration ID CRD42015029349 [19].

### Objectives

We will develop, externally validate and update separate prediction models for (i) early (< 34 weeks’ gestation), (ii) late (≥ 34 weeks) and (iii) any onset pre-eclampsia.

Primary

1. To estimate the prognostic value of individual clinical, biochemical and ultrasound markers for predicting pre-eclampsia by IPD meta-analysis
2. To validate, and improve or tailor the performance of existing models in relevant population groups, for predicting early, late and any onset pre-eclampsia in our IPD dataset based on:

- Clinical characteristics only
- Clinical and biochemical markers
- Clinical and ultrasound markers
- Clinical, ultrasound and biochemical markers

3. Using IPD meta-analysis, to develop and externally validate (using internal-external cross-validation) multivariable prediction models for early, late and any onset pre-eclampsia in the following circumstances: where existing predictive strategies cannot be adjusted for the target population, or where no such models exist for the relevant pre-eclampsia outcomes.

Secondary

4. To assess the differential performance of the existing models in various predefined subgroups based on population characteristics (unselected; selected) and timing of model use (first trimester; second trimester)
5. To study the effect on accuracy of adding novel metabolic and micro-RNA based biomarkers to the developed model based on clinical, ultrasound and biochemical markers

### Literature search

We have previously undertaken the relevant systematic reviews on clinical characteristics, biochemical and ultrasound markers for prediction of pre-eclampsia [20–34]. As a first step in the IPD meta-analysis, we will undertake a systematic review of reviews, and additionally search for primary studies not included in existing reviews, as new research evidence may have appeared since completion of our work. We will also update our systematic review of prediction models for pre-eclampsia [35] by searching the following databases: MEDLINE, EMBASE, BIOSIS, LILACS, Pascal, Science Citation Index, Cochrane Database of Systematic Reviews (CDSR), Cochrane Central Register of Controlled Trials (CENTRAL), National Institute of Child and Human Development Data and Specimen Hub (NICHD - DASH), Database of Abstracts of Reviews of Effects (DARE) and Health Technology Assessment Database (HTA). Research reported in the grey literature will be sought by searching a range of relevant databases including the Inside Conferences, Systems for Information in Grey Literature (SIGLE), MotherChild Link Registry (http://www.linkregistry.org/search.aspx), Dissertation Abstracts and Clinical Trials.gov. Internet searches will also be carried out using specialist search gateways, general search engines (such as Google: http://www.google.co.uk/) and meta-search engines (such as Copernic: http://www.copernic.com/). Language restrictions will not be applied to the electronic searches. We will further ask primary authors to examine the included study list to identify any studies, birth cohorts or datasets that may have been missed. Collaborative groups such as The Global Pregnancy CoLaboratory (CoLab), Pre-eclampsia and Eclampsia Monitoring, Prevention and Treatment (PRE-EMPT) and Global Obstetrics Network (GONet) will also be approached to identify primary studies, unpublished research and birth cohorts [36–38].

### Establishment of the IPPIC pre-eclampsia (International Prediction of Pregnancy Complications) Network

We have established a collaborative network of investigators (IPPIC) from research groups that have undertaken studies on clinical characteristics, biochemical and ultrasound markers in the prediction of early and any onset pre-eclampsia. The network includes 72 researchers from 21 countries. A project-specific website will be developed to improve visibility and communication. A memorandum of understanding will cover the provision of data by the principal investigators of the individual studies. We will agree on a timetable and publication policy (policy of collaborative/group authorship will be confirmed).

### Eligibility criteria for relevant cohorts and studies

All identified primary studies (prospective and retrospective cohort studies, as well as cohorts nested within randomised trials), and large birth and population based cohorts which provide information to assess the accuracy of clinical, biochemical, and ultrasound predictors in low, high or any risk women to predict early, late or any pre-eclampsia and its complications will be eligible for inclusion. Table 1 lists the characteristics of population, predictors and outcome that will be included in the IPD meta-analysis. The predictors will be clearly defined and standardised, and will be chosen a priori for consideration in the evaluation based on the most promising predictor variables. [20–34] The primary outcomes are early (< 34 weeks), late (≥ 34 weeks) and any pre-eclampsia. Pre-eclampsia is defined as new onset hypertension after 20 weeks gestation (BP greater than or equal to 140/90 mmHg) and new onset proteinuria of 2 or more on standard urinary dipstick tests and proteinuria on spot urine PCR (protein creatinine ratio) test greater than 30 mg/mmol or 24 h urine > 300 mg/24 h [39]. The secondary outcome will be a composite adverse maternal or foetal outcome.

**Table 1:** Characteristics of population, predictors and outcome in the IPD meta-analysis on prediction of pre-eclampsia

Population	Pregnant women
Predictors	*Maternal clinical characteristics at booking:* Maternal characteristics: Age, BMI, height, weight, ethnicity, smoking, alcohol or substance misuse; Medical history: pre-existing chronic kidney disease, heritable thrombophilias, autoimmune disease such as systemic lupus erythematosus (SLE) and anti-phospholipid syndrome, type 1 and 2 diabetes and hypertensive diseases; Previous obstetric history: parity, previous pre-eclampsia, GDM, pregnancy interval more than 10 years, family history of pre-eclampsia, family history of cardiovascular disease, previous miscarriages, preterm birth, stillbirth or small for gestational age foetus; Current pregnancy: Multiple pregnancy, mode of conception, early pregnancy bleeding, MAP, systolic and diastolic blood pressure, SES, new partner, diet or exercise in pregnancy, urine dipstick, PCR, 24 h protein *Biochemical markers (first or second trimester):* PAPP-A, sflt-1, PlGF, AFP, HCG, soluble endoglin, CRP, Hyper triglyceridaemia and PAI-1 *Ultrasound markers (first or second trimester):* CRL, AC, expected foetal weight centile, Uterine and umbilical artery Doppler (resistance index, pulsatility index, unilateral or bilateral notching)
Outcomes	*Primary outcomes:* Early (<34 weeks), late (≥34 weeks) and any pre-eclampsia *Secondary outcomes:* *Maternal complications:* Eclampsia, HELLP syndrome, abruption, hepatic and renal failure, cortical blindness, pulmonary oedema, postpartum haemorrhage, DIC, preterm delivery, admission to HDU, maternal death, caesarean section, GDM *Foetal and neonatal complications:* Birth weight in Kg and centile, small for gestational age foetus, stillbirth, neonatal death, hypoxic ischemic encephalopathy, respiratory distress syndrome, septicaemia, admission to neonatal unit

### Study selection, IPD collection and harmonisation

The minimum data to be collected for IPD meta-analysis will be agreed at the first collaborators’ workshop by discussion with the collaborative group. We will contact the authors of primary studies, and datasets to obtain IPD, in any format, along with data dictionaries or descriptions. The data will be obtained in an anonymised format and stored in a secure data repository. All variables recorded, even those not reported in the published studies, will be considered for collection and for planning subgroup analyses with sufficient statistical power. We will build on existing efforts undertaken in standardising the variables in the IPD meta-analysis projects on the prediction of pre-eclampsia, in specific subgroups of women, such as those with a previous history of pre-eclampsia and for particular tests such as uterine artery Doppler ultrasound in the second trimester.

Access to the existing IPD datasets will allow us to rapidly set-up the database for the proposed project. Researchers will supply data in the format most convenient to them. The project team will take responsibility for converting the data to the required format. There will be flexibility in the format and method of transfer of primary data. All data supplied will be subjected to range and consistency checks. Any missing data, obvious errors, inconsistencies between variables or outlying values will be queried and rectified through input from the original authors. At the time of submission of the protocol, we have access to 74 IPD from 72 researchers. These need further cleaning, quality assessment of the study, data quality checks, and assessment of availability of relevant data to evaluate their inclusion in the analysis. The predictors of the original dataset will be matched with the variables in the IPD, and where a direct match is not available in the data, a new variable will be created from other information contained within the original dataset if possible, such as calculating BMI from weight and height, or deriving mean pulsatility index by averaging the left and right pulsatility index measurements.

Missing data over 10% for each variable, range checks for variables with continuous measures, obvious errors, inconsistencies between pre-identified variables that are considered essential for the project or outlying values will be queried and rectified with input from the original authors. We will send two reminders to the original author for response to queries, after which a decision will be taken by the project team on whether to exclude the variable in question or the entire data itself.

We will use existing information within the provided dataset where possible, to obtain information when not available. For example, we shall use weight and height data to calculate BMI. Where there is more than one measurement, we will choose the first measurement. We will consider predictors collected between 0 and 14 weeks as first trimester, > 14–28 weeks as second trimester and >28 weeks as third trimester values. We will also obtain information on treatment such as aspirin, calcium and vitamin supplement use, which could influence the outcome. Although datasets may contain additional variables, we will prioritise acquisition of those that were included in the published prediction models to validate and harmonise (e.g. transform to the same scale or measurement unit if necessary).

### Quality assessment

The risk of bias in individual studies or datasets will be assessed by an early version of the Prediction study Risk of Bias Assessment Tool (PROBAST) [40, 41]. Criteria considered will include participant selection (adequate description of data sources, details on recruitment), predictors (appropriately defined, assessed blinded to outcome, assessed in the same way for all participants), and outcomes (appropriately defined and determined in a similar way for all participants, predictors excluded from the outcome definition, outcome determined without knowledge of predictor information and appropriate interval between assessment of predictor and outcome determination). Applicability of the studies or datasets will also be evaluated using the same tool above. We will assess the extent to which the dataset provided is able to answer the IPD meta-analysis question, in terms of the population and outcomes of interest.

### Data synthesis

In accordance with PRISMA-IPD, a flow diagram will be drawn up showing the number of studies identified through to the number of studies and participants included in the analysis.

For all individual studies used to validate any of the prediction models, study level characteristics will be summarised and presented in Tables. A summary will also be provided for the prediction models to be validated using the collected IPD.

#### Summarising the overall predictive accuracy of individual predictors of pre-eclampsia

Meta-analysis will be used to summarise the prognostic value of each clinical, biochemical, and ultrasound marker, in relation to each of the binary outcomes of early, late and any pre-eclampsia. The markers to be evaluated are based on our systematic reviews in this area. For each of the outcomes and markers of interest, we will perform a two-stage IPD meta-analysis of the prognostic effect, unadjusted and adjusted for particular variables available across studies. The two-step approach first involves fitting a logistic regression model for each study, and then pooling the log odds ratios using a conventional random effects meta-analysis. The random effects model allows for heterogeneity between studies, and will be estimated using REML. The 95% confidence interval for the pooled effect will be derived using the Hartung-Knapp approach. Heterogeneity will be summarised using the *I* ^2^ statistic (which provides the proportion of total variability that is due to between-study heterogeneity) and 95% prediction intervals. The trend across multiple categories and across variables that are continuous will be considered linear, although suitable transformations (e.g. natural log) will be considered if it improves model fit. Only singletons will be included in the analysis, and complete case analysis will be employed.

#### Identifying relevant data for validation of existing models

Each model will be validated using IPD from studies that contain all of the predictors in the model and the relevant outcome (early, late or any pre-eclampsia). Ideally, the time of measurement of the predictors and outcomes should match for the setting in which the model was developed, with generalisability to other measurement times and outcomes assessed later. However, time of predictor and outcome measurement may not always be available, or may differ only slightly. Therefore, a broad inclusion criterion will be used initially, and then subgroups of datasets (e.g. those at low risk of bias) will be considered that match the original model most correctly. Validation performance will be calculated for each individual study separately, rather than using a combined dataset containing all IPD. Model performance will then be summarised across studies using random effects meta-analysis.

#### Missing data

##### Missing predictors

If a predictor from a prediction model is not present within an individual study (i.e. not recorded for any of the participants in that study), this is considered to be systematically missing. Though it may be possible to impute values for the missing predictor based on the IPD from other studies [42–44], for practical reasons, imputation will not be performed for systematically missing variables. Instead only the studies that recorded all predictors for a particular model will be used for validation of a particular prediction model.

If some participants are missing values for predictors within an individual study, multiple imputations will be used to recover data rather than dropping these participants from the analysis as in a complete case analysis. The multiple imputations will be based on the individual study, not the collection of all IPD studies. The imputation process will be performed before any of the analysis takes place, therefore all relevant predictors (for all prediction models to be validated) will be identified and imputed for at the same time to avoid imputing values for each different prediction model separately. This will ensure a coherent set of imputed datasets, to be used consistently in all analyses, regardless of the prediction model being validated. The interest here is performance statistics, which is sensitive to the type of imputation model [45]. The imputation model will therefore include other variables available within the dataset. Using the rule of thumb that the number of imputed datasets (*m*) should be at least equal to the percentage of incomplete observations [46], *m* will be set equal to the largest percentage of incomplete observations in any of the studies, and the same *m* will be used for all studies. For example, if the largest percentage of incomplete observations in any of the studies was 40%, 40 datasets will be imputed for each study. For each validated model, performance statistics (discussed later) will be averaged across imputations using Rubin’s rules to obtain one estimate and standard error (SE) for each performance statistic in each study [47]. This will be done on the logit scale for the C-statistic, as it is unlikely to be normally distributed on the original scale. Within-imputation SEs can be obtained on these transformed scale by applying the delta-method and using the formulae given by Debray et al [48].

Predictors such as previous history of pre-eclampsia may appear missing in some participants, solely because the woman has not previously been pregnant. We will therefore group women into three categories; Multiparous with previous history of pre-eclampsia, Multiparous and no previous history of pre-eclampsia and nulliparous and treat these categories as separate predictors.

##### Missing outcomes

If in an individual study some participants are missing details about whether the outcome occurred or not, even after checking with the original study authors, then these values will be imputed in the same way as missing predictor values, using as many variables as possible (including other available variables in addition to model predictors) in the imputation model. Imputed outcomes will be used in the analyses, rather than deleting observations with missing outcomes [49].

### Other considerations relating to the collected IPD for external validation

#### Women with multiple pregnancies

Patients may be included in a study more than once if they had more than one pregnancy. For the purpose of external validation, we will validate the model for each pregnancy of each patient (i.e. keep all data) and consider each women’s pregnancy as a distinct observation. Though two or more pregnancy outcomes from the same women are likely to be correlated, the number of multiple pregnancies is expected to be very small relative to the total number of pregnancies; further, external validation aims to ensure that a prediction model is accurate for all applications, regardless of whether it was applied to the same women previously.

#### Variables reported using multiples of the median

Some biomarkers and ultrasound markers have large variability due to factors such as gestational age and ethnicity, and vary across laboratories in terms of their method of measurement. Therefore, some researchers report them as multiples of the median (MoM). The MoM of a predictor value for a particular patient is calculated by a laboratory using their own approach. Typically this is based on comparing the predictor value for that patient against the median value in that laboratory’s population, often after adjustment for other factors (e.g. gestational age and ethnicity). Unfortunately, different laboratories may adjust for different factors when calculating MoMs of a predictor, and even if the same adjustment terms are used, the magnitude of the adjustment effects (adjustment equation) is not necessarily consistent across laboratories.

Several of the prediction models to be validated include biomarkers and/or ultrasound features reported as MoMs, but not all IPD studies report the predictors as MoMs. Such models will be validated only using those studies that have reported MoMs for those predictors. We will not calculate MoMs for IPD studies that do not directly report the predictors as MoMs. This is because we do not know what factors the other laboratories would have adjusted for, how the adjusted medians would be obtained, and what the laboratory set medians would be. It would only be possible to calculate medians for the patients within that study, rather than any larger population, and thus would have not represented actual practice. It was also not practical to contact the many laboratories represented in this IPD obtained.

Biomarkers may be measured using different assays and platforms. As such, we will adjust for the biomarker assay and platform in our analysis, and will consider these as separate variables in our models.

#### IPD studies that do not report gestational age for pre-eclampsia diagnosis

If a study does not report the gestational age when pre-eclampsia is diagnosed, there is a possibility of the outcome occurring prior to biomarker measurement in some studies. If gestational age at diagnosis of pre-eclampsia is not recorded, we will use gestational age of delivery as a proxy for gestational age of diagnosis.

### External validation performance of existing models

If any of the studies for which IPD have been collected were already used to develop a prediction model, it will be excluded from the studies used to validate that particular model. This is because performance would be over-optimistic in that dataset and will provide only apparent or internal validation performance of the model rather than external validation performance, which is of interest to the IPD project.

We will report the predictive performance of a model in terms of discrimination and calibration. Calibration refers to how well the predictions from the model agree with the observed outcomes, while discrimination relates to how well a model can separate between women that develop pre-eclampsia and those that do not [50, 51]. The performance statistics are defined below, and will be calculated for each study separately, using (at least initially) all relevant participants in each study. These will then be summarised across studies using meta-analysis methods.

For each prediction model in each individual study, the model equation will be applied to each participant in the IPD to calculate the linear predictor value for that participant (*LP*_*i*_, value of the linear combination of predictors in the model equation for individual *i*), as well as the predicted probability of pre-eclampsia (using the inverse logit transformation of *LP*_*i*_).

For each prediction model, the distribution of *LP*_*i*_ values will be summarised for each study. The following validation performance statistics will then be calculated:

#### C-statistic (discrimination)

The concordance statistic (C-statistic) gives the probability of a randomly selected woman with pre-eclampsia having a higher predicted probability than randomly selected women without pre-eclampsia. The C-statistic is equivalent to the area under the ROC curve, and will be calculated (along with its SE) using non-parametric ROC analysis in Stata using the ‘roctab’ command. It is likely that the distribution of the C-statistic is not normal since it is a proportion and therefore bounded by the value 1. Therefore the logit scale will be used to pool across imputations (as this is also the scale that will be used later in the meta-analysis) [52]. The SE for logit(C-statistic) can be calculated from the C-statistic and SE for the C-statistic using the following formula [48]:

$$ \mathrm{SE}\left(\mathrm{logit}\left(\mathrm{C}\right)\right)=\frac{\mathrm{SE}\left(\mathrm{C}\right)}{\mathrm{C}\left(1-\mathrm{C}\right)} $$

#### Calibration-in-the-large

This measure indicates the extent that model predictions are systematically too low or too high across the dataset. The estimate of calibration-in-the large and its SE will be calculated by fitting the calibration model logit(*p*_*i*_) = *α* + *β*(*LP*_*i*_) where *α* is the estimate of calibration-in-the-large, when *β* = 1 (fitted using an offset term) and *i* refer to a participant.

#### Calibration slope

The calibration slope indicates whether there is agreement between observed outcomes and predictions across the range of predicted risks. The calibration model, logit(*p*_*i*_) = *α* + *β*(*LP*_*i*_) will be fitted and $\widehat{\beta}$ is the estimated calibration slope. Ideally, the calibration slope would be equal or very close to 1 for good calibration. However, a slope < 1 indicates overfitting of the model, whereas a slope > 1 indicates underfitting.

#### Calibration plots

A graph showing the observed (O) and expected (E) probabilities for groups of patients. Patients will be grouped into deciles of the predicted probability, and O versus E given for each group. A lowess smoother will be applied to show the overall calibration slope, as calculated using all participants. As calibration plots cannot be pooled across imputations, a calibration plot will be drawn for each imputed dataset [53]. If the plots look similar across imputations, the calibration plot from one imputed dataset will be reported to illustrate this. If different patterns are observed in different imputed datasets, then a selection of plots may be presented.

### Summarising model performance

Meta-analysis methods will be used to summarise a model’s performance across all IPD used for external validation. Random-effects will be used rather than fixed-effect meta-analysis because it seems reasonable that the performance of a model may differ across populations due to case-mix [48, 54]. Random-effects meta-analysis will also allow us to quantify any heterogeneity in performance across studies and predict model performance in other similar settings using approximate 95% prediction intervals [55]. The random-effects model for a performance measure can be written as

$$ {Y}_k\sim \mathrm{Normal}\left({\mu}_k,{\upsigma_k}^2\right) $$

$$ {\mu}_k\sim \mathrm{Normal}\left(\mu, {\tau}^2\right) $$

where *k* refers to the study. The model assumes normality of the within-study and between-study performance statistic. Based on the results of a simulation study [52], the C-statistic will be pooled on the logit scale, as the simulation study suggested this to be a more appropriate scale for pooling C-statistics in a meta-analysis. The calibration slope and calibration-in-the large will be pooled on their original scale. Model performance will be summarised for each statistic as the average and 95% confidence interval for the average performance statistic. Confidence intervals will be derived using the Hartung-Knapp approach to account for uncertainty in variance estimates [56]. Heterogeneity in model performance across studies will be summarised using the estimates of *I* ^2^ statistic [57], and *τ* ^2^, with approximate 95% prediction intervals calculated using the approach of Higgins et al. [58].

Model performance across studies will also be shown graphically using forest plots for each performance statistic and scatter plots to show measures of calibration and discrimination in combination (to give an idea of overall performance of the model).

### Additional analyses relating to validation performance of prediction models

#### Risk of bias

Performance of each prediction model (as described above) will also be summarised according to the risk of bias (using PROBAST) where there are enough studies to do so; for example, summarising model performance statistics for only the studies that are low risk of bias for specified criteria to assess whether there is less heterogeneity in performance.

#### Generalisability of the model

Further analyses may include evaluating how widely the model can be applied and how this affects the model performance, each model could be applied to the following settings:

- Different timing of the outcome (e.g. any pre-eclampsia if the model was developed to predict early pre-eclampsia)
- Different time of predictor measurement

##### Meta-regression

If there are enough studies in the analysis (10 or more studies), we will consider meta-regression models as an exploratory analysis to investigate if there are any differences in the performance statistics due to the following pre-defined study-level factors: outcome definition, study design, timing of the outcome, timing of the predictor measurement, method of measurement of predictor values, mean linear predictor, and variability of linear predictor (such as standard deviation).

### Subgroup analyses

If a specific model performs reasonably well, say with a C-statistic comparable or greater than that of the other prediction models, and a calibration slope between 0.9 and 1.1 on average across validation studies, we may interrogate the model performance further within specific subgroups. For example, in key patient groups such as groups defined by age, parity and BMI. Meta-analysis will be used to summarise subgroup performance across studies where appropriate.

### Publication and related biases

Publication bias is not expected because IPD are being collected independent to the external validation performance of each model. However, if there are 10 or more studies for a particular model, then we will examine whether there are small-study effects (potential publication bias) on contour-enhanced funnel plots. If there are small-study effects, the funnel plot will be asymmetric, with larger studies showing different performance estimates than smaller studies. Publication bias, IPD selection bias and IPD availability bias may be underlying reasons for any asymmetry. However, we will recognise that heterogeneity may also be a genuine reason, for example with smaller studies coming from populations or cohorts with different case-mix variation.

### Comparison of the performance of different models

If there is a subset of multiple studies that contain all the predictors of two or more models that appear to have good predictive performance upon validation (based on the meta-analysis), then we will use this subset of studies to directly compare the performance of these models. Models will be ranked according to their discrimination (largest C-statistic) and calibration measures such as calibration slope. If there are enough studies available, a bivariate meta-analysis of the C-statistic and the calibration slope will be performed to jointly summarise discrimination and calibration performance. The results of the meta-analysis can be used to calculate the probability of ‘good’ performance in future settings in terms of both discrimination and calibration, where a good C-statistic is defined as C-statistic ≥ 0.7 and good calibration defined as a calibration slope between 0.9 and 1.1 [59].

Decision curve analysis is another method for evaluating and comparing prediction models (in addition to the traditional validation measures of calibration and discrimination). The net benefit of the model is plotted against different probability thresholds to produce a decision curve [60]. To obtain the curve, the prediction model is evaluated at different probability thresholds where the threshold is taken as a point above which a patient would be treated, and below which a patient would not be treated. The curve can then be compared against the treat all and treat no-one strategies to see the range of probabilities at which the model may be useful. Decision curves can also be plotted for different models on the same graph for comparison, and to help decide which model offers the most benefit.

Decision curve analysis will be used to show the net benefit of the pre-eclampsia prediction models being externally validated, again using the subset of studies for which a direct comparison of the most promising models is possible. Decision curve analysis will be used to compare them and see if one model offers greater net benefit than the other. The model with the highest curve (over a range of thresholds) is considered to have the greatest net benefit.

Decision curve analysis will be run in Stata using the *dca* command [61].

### Updating (recalibrating) existing prediction models

Within each of the model categories (clinical, clinical and biomarker, clinical and ultrasound, all three types of markers), if a prediction model can be identified which has good average discriminative performance (C-statistic is comparable to, or greater than that of other models), but is mis-calibrated (calibration slope not between 0.9 and 1.1) or has large heterogeneity in calibration performance across different validation studies, we will consider recalibration techniques such as using study-specific intercepts, in an attempt to improve model performance. If recalibration does not considerably improve the performance of the model, we will consider developing and validating a new prediction model for that model category.

### Developing and validating new prediction models

If no existing model shows good performance even after recalibration, and there is sufficient data to do so, we will consider whether it is possible to develop and validate a new prediction model as necessary. This is dependent on the amount of data available with common variables across studies and on the number of events. Early onset pre-eclampsia is the rarest of the three outcomes (0.5% of all pregnancies). As a rule of thumb when developing a prediction model, we need at least 10 events for each candidate predictor variable to reduce the potential for large overfitting. If necessary, we will limit the number of candidate predictors considered to achieve this. However, we are likely to have an adequate number of events per variable using IPD from multiple studies.

#### Model development framework

If new prediction models are developed, a logistic regression framework will be used as pre-eclampsia is a binary outcome (yes/no). A separate intercept will be used for each study to allow for differences in the baseline risk (e.g. different prevalence levels in different settings). We will also fit a random intercept model and compare the two approaches (stratified intercept versus average intercept) in terms of model performance. We will also consider heterogeneity in the predictor effects to help inform variable selection (a variable with a homogeneous predictor effect across studies is preferred to a heterogeneous one). The same multiply imputed datasets as used in the validation exercise will be used for model development, with Rubin’s rules used to combine parameter estimates across imputations as before. As correlated outcomes per person may affect the standard errors of model parameter estimates, we will check whether an analysis that accounts for multiple pregnancies per woman has any impact. This is unlikely, given the minimal proportion of multiple pregnancies for the same woman.

#### Predictors to consider in model development

We will aim to identify a set of variables that are recorded in several of the studies (aiming for at least five studies). If a variable is only recorded in one or two studies, external validation in the other IPD studies will also not be possible or will be very limited. We also want a model that includes variables that are likely to be routinely recorded in pregnant women.

Some variables are recorded using different scales, such as the original predictor values and MoMs for biomarkers and ultrasound variables, which are problematic to convert to the same scale across studies. In this case, we will look at which scale has been used most commonly in the IPD studies available, and use that scale, to maximise the amount of data available for development and validation.

#### Variable selection methods

Variable selection and deciding the functional form of continuous variables (e.g. linear or non-linear function) will take place within each cycle of the internal-external cross-validation (detailed below). A multivariable fractional polynomial (MFP) approach will be used, in which fractional polynomial functions are tested for continuous variables to determine the ‘best’ functional form of that variable in the multivariable model (i.e. in the presence of other variables rather than the best functional form determined in a univariable model). Variables that are well known to be predictors of pre-eclampsia will be included in the prediction model, regardless of the significance level, as agreed upon a consensus meeting. The MFP approach begins with a full model (includes all potential predictors being considered), and then backward elimination is applied, removing the least significant variable in each cycle of the procedure if the Wald test *p*-value for that variable is greater than a specified value. The criteria for elimination will be *p* > 0.157, which is used as a proxy for selection based on all-subset Akaike’s Information Criteria (AIC) [62]. If data within the studies have been imputed, the imputed datasets will be stacked and a weighting applied to each dataset to perform variable selection [63]. This will be done using the *mfpmi* command in Stata, using tests of Wald statistics for nested hypotheses and the difference in Wald statistics for non-nested hypotheses [64].

#### Internal-external cross-validation

An internal-external cross-validation (IECV) approach has been proposed for model development and validation when IPD are available from multiple studies [53, 65]. Using this approach, a model is developed using all but one study which is reserved for model validation. The model is then internally validated using the same data, and using methods such as bootstrapping to calculate the internal validation performance of the model. If necessary, a shrinkage factor will be calculated and applied to the regression coefficients. This model is then applied to the omitted study and the validation performance statistics calculated again. This process is repeated multiple times, each time reserving a different study for ‘external’ validation.

#### Overall model performance

Following IECV, there will be multiple statistics for each validation performance statistic (one from each study). These estimates will be summarised using random-effects meta-analysis.

All analyses will be carried out using Stata MP 14.2.

## Discussion

There has been an intense research effort to develop clinically useful predictive tests for pre-eclampsia, which remains a major contributor to maternal and perinatal mortality and morbidity. Early identification of those at increased risk of the disease would potentially allow for targeted surveillance and intervention. It would also mean that less intensive antenatal care is offered to those at reduced risk. The current method of risk prediction based on clinical history has limited predictive accuracy, however it has been suggested that the addition of biochemical and ultrasound markers may improve the predictive performance. Given the large number and diversity of the proposed tests, prediction models have proliferated and become too numerous for most researchers and/or healthcare providers to identify the most clinically useful.

Systematic reviews on the performance of tests in pre-eclampsia will need to take into account the variation in population and test characteristics, treatment provided and the timing of onset of pre-eclampsia. Numerous systematic reviews with aggregate data meta-analysis have evaluated various risk factors separately or in combination for prediction of pre-eclampsia. These aggregate data reviews are affected by the following limitations.

Firstly, the aggregate meta-analyses are restricted by the heterogeneity in the characteristics of the population, timing of tests and cut offs, and the definitions of outcome in published studies. This is especially problematic for the relatively rare but clinically important outcome of early onset pre-eclampsia, which is often not reported in individual studies. Heterogeneity in patient selection can be reduced by IPD through strict inclusion and exclusion criteria (i.e. removal and addition of particular patients in the dataset). Improved accuracy of diagnosis and better definition of outcomes, particularly gestational age at onset of pre-eclampsia, could be accessed in individual patients by IPD meta-analysis, and this is not possible when only aggregate data are available.

Secondly, primary studies often report on only one test or prediction model, despite available information on more than one. Furthermore, any information on the performance of multiple predictors in individual studies is provided as mean values for the population. Hence, it is difficult to undertake sensible evidence synthesis by aggregate data meta-analyses, for evaluation of multiple predictors. Furthermore, aggregate data meta-analyses of multiple predictors have limited capabilities to develop prediction models, yielding accurate estimates of absolute risk for individual patients, particularly in the presence of between-study heterogeneity. By accessing the individual data, IPD meta-analysis will provide a much larger sample size to evaluate several candidate prognostic factors in combination and subsequently develop clinically relevant robust models. Access to IPD will also enable recalibration of the prediction models in the presence of between-study heterogeneity, and hence improve the quality of individual risk predictions.

Thirdly, there is a need for appropriate methods of meta-analysis to summarise the factor-outcome associations. Due to numerous problems of published primary studies investigating factor-outcome associations, especially publication bias and selective reporting, aggregate meta-analyses based on published results are notoriously prone to bias, and show inconsistent and even contradictory factor-outcome associations. The PROGRESS group have shown multiple examples, across a broad range of diseases, where aggregate data meta-analysis has failed to identify clear conclusions about prognostic factors [66] due to poor reporting. In IPD meta-analysis, the association between future outcome and patient-level characteristics and study level characteristics (setting, timing, study design) can be assessed more reliably; for example using a more consistent set of adjustment factors and modelling biomarkers on their continuous scale (rather than categorisation) [67].

Fourthly, prior to application of a model in clinical practice, there is a need to evaluate its performance in the population(s) in which it is intended for use. This requires external validation of the model in a dataset different to that in which it was developed, requiring additional sample size beyond model development, and only possible with IPD (as aggregate data does not allow predictions from a new model to be checked at the patient-level). Lack of external validation is one of the key reasons for the models not being adopted in clinical practice. IPD meta-analysis offers an accepted way to overcome this current lack of validation [68]. Further, we will maximise the data for model development and external validation by using an ‘internal-external cross validation’ approach that accounts for multiple studies by rotating them between model development and validation. External validation performance (e.g. in terms of calibration and discrimination) can then be checked in each study, and summarised itself in a meta-analysis [65, 69].

Fifthly, problems with aggregate data arise with differential treatment effects such as use of aspirin, by patient characteristics. Obtaining individual participant data (IPD) from these studies will facilitate a more reliable meta-analysis, as treatment with aspirin will be available at the individual-level. This will allow, for example, the external validation performance of a model to be evaluated across different groups of individuals defined by their treatment, and considering the inclusion of treatment as a predictor in the models.

An IPD meta-analysis framework with access to the predictor-outcome data of individual patients, will allow for the development and validation of multivariable prediction models for early, late, and any pre-eclampsia. Our prediction models will attempt to achieve this in the following ways: use rigorous statistical methods to develop the models and assess accuracy; undertake a formal external validation within the IPD datasets; use unambiguous definitions of predictors and reproducible measurements using methods available in clinical practice; adjust and/or evaluate performance according to current clinical management; involve patient groups in model development and implementation; and produce personalised risk scores that enable mothers and clinicians to make more informed decisions on management aspects such as commencement of aspirin early in pregnancy and frequent monitoring in secondary and tertiary care. The performance of the model will naturally be limited by the strength of the predictive relationships between the measured variables and the outcome.

A good prediction model is one that yields accurate (reliable) and consistent performance; validated in populations and datasets external to those used to develop the model; widely applicable in practice; acceptable to patients and ultimately improves clinical outcomes by helping clinicians and patients make more informed decisions. External validation should ideally be done across all clinical settings and relevant patient subgroups, in relation to the clinical context of definitions for the start point (i.e. when predictions are made from) and endpoint (e.g. early versus late onset pre-eclampsia, or both).

## Abbreviations

AIC: Akaike’s Information Criteria
BP: Blood pressure
CDSR: Cochrane Database of Systematic Reviews
CENTRAL: Cochrane Central Register of Controlled Trials
CoLab: The Global Pregnancy CoLaboratory
DARE: Database of Abstracts of Reviews of Effects
GONet: Global Obstetrics Network
IECV: Internal external cross validation
IPD: Individual participant data
IPPIC: International Prediction of Pregnancy Complication (IPPIC) Network
LP: Linear predictor value
MFP: Multivariable fractional polynomial
MoM: Multiples of the median
PCR: Protein creatinine ratio
PRE-EMPT: Pre-eclampsia and eclampsia monitoring, prevention and treatment
PRISMA-IPD: Preferred Reporting Items for Systematic Reviews and Meta-Analyses for IPD
PROBAST: Prediction study Risk of Bias Assessment Tool
PROGRESS: Prognosis Research Strategy Partnership
PROSPERO: International prospective register of systematic reviews
ROC: Receiver operating curve
SE: Standard error
SIGLE: Systems for Information in Grey Literature
